# Neuropsychiatric Adverse Effects of Trimethoprim-Sulfamethoxazole: A Rare Case of Acute Psychosis in an Immunocompetent Patient

**DOI:** 10.7759/cureus.68700

**Published:** 2024-09-05

**Authors:** Moujib Omri, Mohamed Ferhi, Jihenne Mannai, Khalil Ben Salah

**Affiliations:** 1 Psychiatry and Psychotherapy Department, Klinikum Mutterhaus der Borromäerinnen, Trier, DEU; 2 Psychiatry Department, Mohamed Taher Maamouri University Hospital, Nabeul, TUN; 3 Psychiatry, Ibn El Jazzar University Hospital, Kairouan, TUN; 4 Surgery, Ibn El Jazzar University Hospital, Kairouan, TUN

**Keywords:** brief psychotic disorder, drug-induced psychosis, medication-induced hallucinations, neuropsychiatric side effects, trimethoprim-sulfamethoxazole

## Abstract

Trimethoprim-sulfamethoxazole (TMP-SMX) is a combination of two antibiotics used to treat various bacterial infections, generally well-tolerated but can rarely cause neuropsychological adverse effects, including psychosis. This case report describes a 69-year-old immunocompetent female who developed acute visual and auditory hallucinations three days after starting TMP-SMX for a urinary tract infection (UTI). The patient had a history of depression, successfully treated with mirtazapine a decade ago, and no other psychiatric or medical conditions. Laboratory tests and imaging were unremarkable. Symptoms resolved completely within two days of discontinuing TMP-SMX, suggesting a causal relationship. This case highlights the need for vigilance regarding the neuropsychiatric side effects of TMP-SMX, even in immunocompetent individuals, and underscores the importance of considering medication-induced psychosis in differential diagnoses. Further research is warranted to elucidate the mechanisms underlying this adverse drug reaction.

## Introduction

Trimethoprim-sulfamethoxazole (TMP-SMX) is a combination of two antimicrobial agents effective against a wide range of bacteria and some protozoa. It is commonly used to treat aerobic Gram-positive and Gram-negative bacterial infections, including UTIs, gastrointestinal infections, pneumonia, and cellulitis. In addition, TMP-SMX serves as a first-line agent in the prophylaxis of toxoplasmosis and *Pneumocystis jirovecii *pneumonia [1]. The two components, TMP and SMX, work sequentially to inhibit enzyme systems of the bacterial synthesis of tetrahydrofolic acid [2].

While TMP-SMX is generally well-tolerated, particularly in young and healthy individuals, it can cause common side effects such as nausea, vomiting, rash, pruritus, and hypersensitivity reactions. Less common adverse effects include nephrotoxicity, hepatitis, megaloblastic anemia, and Stevens-Johnson syndrome [1]. It is crucial for physicians to be aware of the drug’s potential neuropsychological adverse effects, which include hallucinations, delusions, depression, agitation, confusion, and suicide attempts, as these symptoms require immediate discontinuation of the medication [3,4]. However, it is important to note that these side effects are rare, and most patients tolerate the medication without significant issues [3].

An uncommon and lesser-known side effect of TMP-SMX is psychosis. There are few case reports in the literature that have documented an association between TMP-SMX and psychosis, predominantly in immunocompromised patients [3-11], given that central nervous system toxicities are uncommon and extremely rare in immunocompetent patients​ [9,12,13].

The uniqueness of the present case lies in the fact that the patient was immunocompetent, highlighting the need for awareness of this potential adverse effect in a broader patient population.

## Case presentation

A woman in her 60s presented to the psychiatric emergency department with acute visual and auditory hallucinations that began three days prior. The patient had a history of depression 10 years ago, which was treated with mirtazapine for 13 months, and has been paraplegic for the past 40 years following a car accident. She had no history of dementia or other medical conditions. She denied using tobacco, alcohol, or illicit substances and reported no recent stressing factors in her lifestyle that could precipitate hallucinations. She had no adverse childhood experiences and reported no history of delirium, hallucinations, suicidal or homicidal ideation, or seizures.

She reported hearing dialogues from unknown people and the sound of her telephone ringing without anyone answering, during the past three days. In addition, she intermittently observed wall deformations. These hallucinations occurred multiple times a day, significantly disrupting her daily activities, yet were not accompanied by delusions.

On examination, her mood was anxious, but she was oriented to person, place, and time, with no signs of memory impairment or mental confusion. Her cognitive functions, including attention, concentration, and judgment, were intact. The patient was hospitalized, and a Mini-Mental State Examination (MMSE) was performed, yielding a score of 28 out of 30, indicating generally preserved cognitive function with minor impairments in attention.

Further questioning revealed that the patient had been prescribed TMP‑SMX 200 mg twice daily for a UTI by her primary care physician. The onset of her symptoms occurred three days after initiating the antibiotic, raising the suspicion of a drug-induced psychotic reaction. Physical examination revealed no abnormalities, and the patient was afebrile with stable vital signs. She reported no symptoms indicative of an *Escherichia coli *infection.

Laboratory results collected on admission showed normal complete blood count (CBC), electrolytes, liver function tests, thyroid-stimulating hormone (TSH), urine analysis, and serum C-reactive protein (CRP) levels (Table 1).

**Table 1:** Laboratory test results of the present case

Tests	Results	Reference
Complete blood count		
Hemoglobin	14.5 g/dL	13.8-17.2 g/dL (men), 12.1-15.1 g/dL (women)
Hematocrit	45%	41-50% (men), 36-44% (women)
White blood cells	6.9 x 10³/µL	4.5-11 x 10³/µL
Platelets	238 x 10³/µL	150-450 x 10³/µL
Serum C-reactive protein	4	< 5 mg/L
Liver function tests		
Alanine aminotransferase	28 units/L	7-56 units/L
Aspartate aminotransferase	23 units/L	10-40 units/L
Alkaline phosphatase	81 IU/L	40-130 IU/L
Bilirubin, total	0.6 mg/dL	0.1-1.2 mg/dL
Albumin	4.3 g/dL	3.4-5.4 g/dL
Kidney function tests		
Creatinine	0.7 mg/dL	0.6-1.2 mg/dL (men), 0.5-1.1 mg/dL (women)
Blood urea nitrogen	16 mg/dL	7-20 mg/dL
Estimated glomerular filtration rate	93 mL/min/1.73 m²	>90 mL/min/1.73 m²
Thyroid function tests		
Thyroid-stimulating hormone	2.3 µIU/mL	0.4-4.0 µIU/mL
Free thyroxine	1.3 ng/dL	0.9-1.7 ng/dL
Urine analysis		
Appearance	Clear	Normal
pH	6.6	4.5-8.0
Specific gravity	1.014	1.005-1.030
Protein	Negative	Negative
Glucose	Negative	Negative
Ketones	Negative	Negative
Blood	Negative	Negative
Leukocyte esterase	Negative	Negative
Nitrite	Negative	Negative

**Figure 1:**
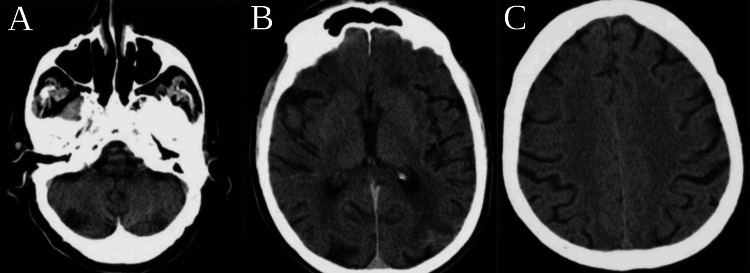
Computed tomography scan of the brain A: axial CT scan at the infratentorial level; B: axial CT scan at the supratentorial level passing through the ventricles; C: axial CT scan at the supratentorial level passing through the frontoparietal lobes.

A comprehensive toxicology screen (Table 2) and a computed tomography (CT) scan of the brain (Figure 1) were performed, both yielding negative results and failing to explain the acute hallucinatory symptoms.

**Table 2:** Toxicology screen results of the present case

Substance	Results	Reference
Alcohol (ethanol)	Negative	Negative
Amphetamines	Negative	Negative
Barbiturates	Negative	Negative
Benzodiazepines	Negative	Negative
Cocaine metabolites	Negative	Negative
Methadone	Negative	Negative
Opiates	Negative	Negative
Phencyclidine (PCP)	Negative	Negative
Tetrahydrocannabinol (THC)	Negative	Negative
Tricyclic antidepressants	Negative	Negative
Acetaminophen	Negative	10-30 µg/mL
Salicylates	Negative	2-20 mg/dL
Lithium	Negative	0.6-1.2 mmol/L
Valproic acid	Negative	50-100 µg/mL

Given the temporal correlation between the initiation of TMP‑SMX and the onset of psychotic symptoms, a decision was made to discontinue the antibiotic, suspecting it as the causative agent. Antipsychotics were withheld to observe if symptom resolution would occur with antibiotic cessation alone. For her anxiety, she was prescribed low-dose lorazepam 2.5 mg daily, administered for three days.

After two days without TMP-SMX, the patient exhibited significant improvement, with complete resolution of auditory and visual hallucinations. She was discharged six days later, asymptomatic for both psychotic symptoms and UTI. Follow-up evaluations in the outpatient psychiatric care confirmed sustained clinical stability. The follow-up period lasted six months, during which the patient remained free of psychotic symptoms and was able to return to her daily activities. Surveillance included regular psychiatric assessments and adherence to up-to-date guidelines for monitoring neuropsychiatric drug reactions. The patient had no recurrence of symptoms, no new psychiatric or medical issues, and continues to live independently. She remains under periodic surveillance to ensure her continued well-being. There have been no reports of her death or any related complications to the described illness.

## Discussion

This case describes a 69-year-old female who presented with acute visual and auditory hallucinations three days after initiating TMP-SMX for a UTI. Upon discontinuation of TMP-SMX, her symptoms resolved completely within two days, suggesting a causal relationship between the antibiotic and the psychosis.

A systematic review [12] published in 2014 investigated antibiotic-associated psychosis during the treatment of UTIs and found that three different classes of antibiotics were implicated in this association, namely, fluoroquinolones, penicillins, and TMP‑SMX. In a study of 1121 hospitalized patients, serious toxicity associated with TMP-SMX was rare, and no episodes of delirium were reported [13]. Although TMP-SMX-induced psychosis is infrequently documented, it has been predominantly observed in elderly [10,11] or immunocompromised individuals [7-9]. However, there are also reports of TMP-SMX-induced psychosis in healthy adults without any psychiatric history. In these cases, the patients experienced visual and auditory hallucinations, with one particularly severe instance resulting in a self-inflicted gunshot wound [3,5,6].

The precise mechanism of TMP-SMX-induced psychosis and central nervous system toxicity remains unclear, but biochemical pathways have been implicated, and TMP-SMX is known for its excellent cerebrospinal fluid penetration [14,15]. Trimethoprim irreversibly inhibits dihydrofolate reductase (DHFR), limiting the conversion of dihydrofolate to tetrahydrofolate, the active form of folic acid. Folic acid deficiencies have been linked to diverse neuropsychiatric sequelae such as dementia, depression, and cognitive impairment [16]. In addition, DHFR is critical for reducing dihydrobiopterin to tetrahydrobiopterin (BH4). A deficiency of BH4, a cofactor in the biosynthesis of biogenic amines [17], has been associated with schizophrenia [18].

The diagnostic criteria for medication-induced psychosis include the presence of delusions or hallucinations that occur during or shortly after the intoxication or withdrawal of a medication. These symptoms must not occur in the context of another psychotic disorder or delirium and must cause significant distress or impairment [19]. The temporal association between TMP-SMX administration and the onset of symptoms, coupled with the resolution of symptoms following drug discontinuation, strongly supports the diagnosis of TMP-SMX-induced psychosis.

The mean half-life of both components of TMP-SMX is between eight and 10 hours, leading to steady-state concentrations within approximately three days [14,15]. This pharmacokinetic profile suggests that the mental status changes observed in the patient were likely due to the increased levels of TMP-SMX at a steady state. The patient's symptoms abated two days after the drug was discontinued. This timeline aligns with literature indicating that most cases of TMP-SMX-induced psychosis begin within three days of drug initiation and resolve within 24 to 48 hours [9].

Medical conditions that might have triggered this patient’s hallucinations include dysthyroidism [20], delirium secondary to an infection, and major depressive disorder (MDD). History and physical examination did not reveal signs of dysthyroidism, and TSH levels were normal.

Infections themselves can be associated with acute psychosis. There are case reports of acute psychosis linked to various infectious agents in patients with no known psychiatric history. It is possible that in some cases, psychosis was misattributed to antibiotic treatment rather than the underlying infection [12]. The most well-known association between infection and acute psychosis occurs in geriatric patients with psychosis and a comorbid UTI in the context of either dementia or delirium [21]. However, our patient was fully alert and oriented with preserved cognitive function according to the clinical examination.

Psychosis seen in MDD is typically auditory and mood-congruent, with themes of deserved punishment or hopelessness, and rarely includes mood-incongruent visual hallucinations as seen in this case [19]. The patient’s depression had been effectively managed with mirtazapine, without recurrence of depressive episodes for the past ten years. Furthermore, she had no history of psychosis or other psychiatric illnesses.

While this case report provides valuable insights into the potential for TMP-SMX to induce psychosis in immunocompetent patients, it has several limitations. First, the single-case nature of the report limits the generalizability of the findings. The absence of a control group makes it challenging to definitively establish a causal relationship between TMP-SMX and the psychosis observed. In addition, the patient's long-term paraplegia and history of depression, although well-managed, might have contributed to a unique physiological or psychological state that influenced her response to the medication. Furthermore, the lack of direct biochemical evidence linking TMP-SMX to the psychotic episode restricts our understanding of the exact mechanisms involved. Finally, follow-up was limited to six months, and longer-term outcomes were not assessed, which could provide more comprehensive insights into the recurrence and management of such adverse effects. Future studies with larger sample sizes and longer follow-up periods are needed to confirm these findings and elucidate the underlying mechanisms.

## Conclusions

This case highlights the critical need for clinicians to be vigilant about the potential for TMP-SMX to induce acute psychosis, even in immunocompetent patients with no prior psychiatric history. The temporal association between the initiation of TMP-SMX and the onset of psychotic symptoms, coupled with their resolution upon discontinuation of the antibiotic, strongly suggests a drug-induced cause. This underscores the importance of considering medication-induced psychosis in the differential diagnosis of new-onset hallucinations or delusions. Furthermore, discontinuing the suspected medication should be prioritized over initiating antipsychotics, as this approach can effectively resolve symptoms.
